# Practical kidney dosimetry in peptide receptor radionuclide therapy using [^177^Lu]Lu-DOTATOC and [^177^Lu]Lu-DOTATATE with focus on uncertainty estimates

**DOI:** 10.1186/s40658-021-00422-2

**Published:** 2021-11-13

**Authors:** Peter Frøhlich Staanum, Anders Floor Frellsen, Marie Louise Olesen, Peter Iversen, Anne Kirstine Arveschoug

**Affiliations:** Department of Nuclear Medicine and PET-Centre, Aarhus University Hospital, Palle Juul-Jensens Boulevard 165, 8200 Aarhus N, Denmark

**Keywords:** PRRT, Peptide receptor radionuclide therapy, Kidney dosimetry, Lu-177, Lu-DOTATOC, Lu-DOTATATE, Uncertainty analysis

## Abstract

**Background:**

Kidney dosimetry after peptide receptor radionuclide therapy using ^177^Lu-labelled somatostatin analogues is a procedure with multiple steps. We present the SPECT/CT-based implementation at Aarhus University Hospital and evaluate the uncertainty of the various steps in order to estimate the total uncertainty and to identify the major sources of uncertainty. Absorbed dose data from 115 treatment fractions are reported.

**Results:**

The total absorbed dose with uncertainty is presented for 59 treatments with [^177^Lu]Lu-DOTATOC and 56 treatments with [^177^Lu]Lu-DOTATATE. For [^177^Lu]Lu-DOTATOC the mean and median specific absorbed dose (dose per injected activity) is 0.37 Gy/GBq and 0.38 Gy/GBq, respectively, while for [^177^Lu]Lu-DOTATATE the median and mean are 0.47 Gy/GBq and 0.46 Gy/GBq, respectively. The uncertainty of the procedure is estimated to be about 13% for a single treatment fraction, where the absorbed dose calculation is based on three SPECT/CT scans 1, 4 and 7 days post-injection, while it increases to about 19% if only a single SPECT/CT scan is performed 1 day post-injection.

**Conclusions:**

The specific absorbed dose values obtained with the described procedure are comparable to those from other treatment sites for both [^177^Lu]Lu-DOTATOC and [^177^Lu]Lu-DOTATATE, but towards the lower end of the range of reported values. The estimated uncertainty is also comparable to that from other reports and judged acceptable for clinical and research use, thus proving the kidney dosimetry procedure a useful tool. The greatest reduction in uncertainty can be obtained by improved activity determination, partial volume correction and additional SPECT/CT scans.

## Introduction

Peptide receptor radionuclide therapy (PRRT) using somatostatin analogues labelled with radionuclides has become an established option for treatment of somatostatin receptor positive neuroendocrine tumors (NETs) [1–4], and PRRT is now recommended as second-line treatment for gastro-intestinal NETs [5]. For several years, the standard treatment schedule using the isotope ^177^Lu has been four fractions of 7.4 GBq [^177^Lu]Lu-DOTATATE [6] or to lesser extent [^177^Lu]Lu-DOTATOC [7, 8], but in recent years personalized treatments allowing for an increase of cumulated activity, in anticipation of improved treatment effect, have attracted much attention [9–11], and controlled clinical trials are ongoing [12–14]. This attention is due to the low risk of toxicity with the standard schedule and, in dosimetry studies, the findings of absorbed kidney doses generally being well below dose limits derived from external beam radiation therapy (EBRT) and with large inter-patient differences [15–19].

The kidneys are considered organs-at-risk in PRRT [15], and kidney dosimetry plays a key role in individualized treatments seeking to reach a pre-defined absorbed dose limit to the kidneys [12–14]. The dosimetry implementation should be accurate and reproducible, both in order to compare studies between sites, to relate to dose limits derived from EBRT, where the absolute dose is accurately measured with reference to calibrated ion-chambers [20], and eventually to establish reliable dose limits on the basis of PRRT studies.

Kidney dosimetry is based on a time-activity curve, established by sequential quantitative imaging of gamma-radiation and determination of the ^177^Lu activity in the kidneys [21, 22]. A variety of implementations have been reported, which may vary in several aspects including: Method of SPECT/CT scanner calibration, the use of planar imaging, 3D imaging or a hybrid thereof, the number of scans performed after therapy, and their time points, as well as the method of kidney delineation and correction for partial-volume effects [21, 23, 24]. A given implementation will introduce random and systematic effects, which should be evaluated in order to ensure comparable measures of absorbed dose. To this end, publications on standardized dosimetry, uncertainty evaluations and site-to-site comparisons are of great importance [21, 24–28].

In this paper we present the implementation of kidney dosimetry at our institution, evaluate the uncertainty of the various steps in the process and summarize the total uncertainty in an uncertainty budget. The purpose is both to identify the major sources of uncertainty and to obtain an estimate of the total uncertainty. The uncertainties are estimated from measurements, experience or available literature. The absorbed dose and the specific absorbed dose (absorbed dose per injected activity) to the kidneys of patients treated with either [^177^Lu]Lu-DOTATATE or [^177^Lu]Lu-DOTATOC in the period November 2015 to January 2021 are reported, and the specific absorbed doses are compared to the results from other sites.

## Methods

### PRRT treatment and patient cohort

PRRT was performed using either [^177^Lu]Lu-DOTATATE (Lutathera®; Advanced Accelerator Applications and later Novartis) or in-house produced [^177^Lu]Lu-DOTATOC.

[^177^Lu]Lu-DOTATOC was produced using no-carrier-added [^177^Lu]LuCl_3_ provided by ITG (Isotope Technologies Garching, Germany) on a semi-automatic synthesis unit, Modular-Lab PharmTracer from Eckert & Ziegler. The radiolabeling was achieved by heating the DOTATOC (230 µg per 10 GBq until march 2020 and 115 µg per 10 GBq thereafter) to 80 °C in the presence of [^177^Lu]Lu^3+^ in 20 min. The pH was kept between 4.0 and 4.5 using an acetate/ascorbate-buffer. After radiolabeling the product was purified using a Sep-Pak C18 cartridge, rinsed with saline and released with ethanol through a sterile filter. To the product vial was added ascorbic acid (150 mg/0.85 mmol) and DTPA (20 mg/0.04 mmol), this addition was performed prior to radiolabeling. The final product contained [^177^Lu]Lu-DOTATOC 5.0–21.8 GBq (from march 2020 up to 50.1 GBq) in a volume of 17–19 ml. From the product vial the requested activity was drawn into a syringe and if necessary diluted with saline to reach a minimum volume of 10 ml.

[^177^Lu]Lu-DOTATATE (Lutathera®) was provided with 7400 MBq at planned time of infusion in a volume of 20.5–25.0 ml. The peptide amount was 10 µg/ml [29].

[^177^Lu]Lu-DOTATATE or [^177^Lu]Lu-DOTATOC was injected through a peripheral or central venous catheter using a manually operated infusion system, and the full infusion was given in 5–10 min including flushing with saline.

For protection of the kidneys an infusion of Vamin-18 (Fresenius Kabi AG, Bad Homburg, Germany) or a solution containing 2.5% arginine and 2.5% lysine dissolved in 1 L of saline [3, 30] was infused. Vamin-18 was used from the first treatment in November 2015 and until June 2016 when the arginine/lysine mixture became available for purchase in Denmark. Infusions were given in one of three different protocols according to the kidney function determined by glomerular filtration rate (GFR) measurement normalized to body-surface area using [^51^Cr]Cr-EDTA or [^99m^Tc]Tc-DTPA and according to risk factors such as diabetes, hypertension or previous radionuclide therapy. Antiemetics, anti-inflammatory drugs and pain-relievers were given together with the infusions. Table 1 provides an overview of these protocols and the selection criteria. Pre-therapeutical standard GFR < 30 ml/min/1.73 m^2^ was an exclusion criterion for PRRT.

**Table 1:** Nephroprotection protocols in PRRT and selection criteria

Protocol	Criteria	Infusion of Vamin-18 (before June 1st 2016)	Infusion of arginine/lysine mixture (after June 1st 2016)
A^a^	Pre-therapeutical standard GFR ≥ 50 ml/min/1.73m^2^	1 l solution over 4 h starting ½ hour pre-treatment	1 l solution and 1 l saline over 4 h starting ½ hour pre-treatment
B	Pre-therapeutical standard GFR of 40–49 ml/min/1.73m^2^, previous PRRT or multiple risk factors (*e.g.* nephrotoxic chemotherapy, longstanding diabetes or hypertension)	3 l solution over 12 h starting ½ hour pre-treatment	2 l solution and 1 l saline infused over 12 h starting 1 h pre-treatment. Additionally ½ l solution infused over 2 h starting 24 h post-treatment
C	Pre-therapeutical standard GFR of 30–39 ml/min/1.73m^2^	4 l solution over about 24 h starting ½ hour pre-treatment (150–170 ml/h)	3 l solution and 1–2 l saline infused over 24–36 h starting 1 h pre-treatment. Additionally ½ l solution infused over 2 h starting 48 h post-treatment

^a^Infusion of the arginine/lysine mixture in Protocol A corresponds to the EANM guidelines recommendations [3]

The patients in the cohort included in this paper were given their first treatment fraction with either [^177^Lu]Lu-DOTATATE or [^177^Lu]Lu-DOTATOC in the period from November 2015 to June 2020. Data from additional treatment fractions of these patients were included until January 8 2021. [^177^Lu]Lu-DOTATATE (Lutathera®) was prescribed from November 2015 until September 2016, when our in-house produced [^177^Lu]Lu-DOTATOC was approved and ready for patient administration. In November 2018 we switched back to using [^177^Lu]Lu-DOTATATE (Lutathera®), as the drug was listed by EMA/Danish Medicines Agency for use in patients with gastroenteropancreatic neuroendocrine tumors grade 1–2, except for the patients who already had initiated a [^177^Lu]Lu-DOTATOC treatment series. Patients with disease outside the indications for Lutathera® (*e.g.* G3 neuroendocrine neoplasms [31], pulmonary NETs, paraganglioma, pheochromocytoma and meningioma) were treated with [^177^Lu]Lu-DOTATOC also after November 2018. This use was approved by the Danish Medicines Agency after application for individual compassionate delivery permit.

The standard treatment comprised 4 treatment fractions with injection of 7–8 GBq [^177^Lu]Lu-DOTATATE or [^177^Lu]Lu-DOTATOC and normally with 6–12 weeks between consecutive fractions. For some of the patients deviations were observed, *e.g.* cancellation of further treatment fractions due to progression, treatment break due to treatment of other disease/conditions, reduced activity due to risk factors for nephrotoxicity, 2 fractions of [^177^Lu]Lu-DOTATATE or [^177^Lu]Lu-DOTATOC following 2 fractions of [^90^Y]Y-DOTATOC treatment for patients with bulky tumors, or salvage therapy after a period of stable disease followed by progression [32].

For dosimetry of the kidneys, a SPECT/CT scan of the upper abdomen, including the kidneys, was performed the day after each treatment fraction (Day 1) and additionally after one fraction also on Day 4 and Day 7 for determination of the effective decay rate of ^177^Lu in the kidneys, following the strategy of *e.g.* the Uppsala [15] and the Rotterdam site [17]. In rare cases the scan at Day 4 or Day 7 was shifted by one day due to patient requests or public holidays.

### Dosimetry procedure and uncertainty estimates

**Fig. 1:**
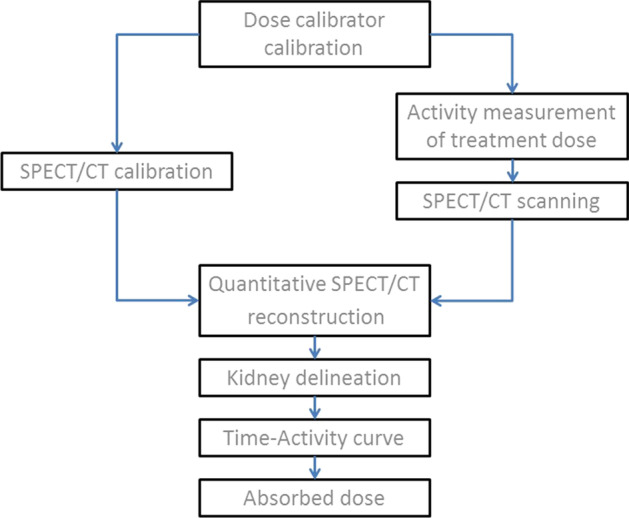
Schematic overview of important steps in the dosimetry procedure

The kidney dosimetry procedure involved multiple steps of calibration, measurement, analysis and calculation. A schematic overview is shown in Fig. 1. Briefly, the calibration of dose calibrators was the starting point for both SPECT/CT scanner calibration and activity measurement of patient doses. Following treatment of the patient, one or more SPECT/CT scans were performed with a quantitative image reconstruction, where the voxel values expressed the activity concentration in each voxel. The images were analyzed in order to generate a time-activity curve for the kidneys to finally calculate their absorbed dose.

In the following we discuss in more detail the individual steps of the dosimetry procedure and the related uncertainties. Uncertainties are stated as the standard uncertainty. For some components the standard uncertainty was estimated from the upper and lower limits of a number of observations, where it was assumed, that the probability of falling within these limits (range 2*a*) was practically equal to one. In these cases a symmetric triangular distribution of width 2*a* was assumed, in which case the standard uncertainty became *a*/6^0.5^ [33]. Standard uncertainties were rounded up to 1% precision.

#### Dose calibrator calibration and activity measurement

In 2015 three dose calibrators (Veenstra VDC-405) were calibrated using a ^177^Lu reference source of 500 µl [^177^Lu]LuCl_3_ (no-carrier-added) in a vial delivered from ITG. Geometry specific calibration factors were determined for (i) delivery vials with small volumes of [^177^Lu]LuCl_3_, (ii) [^177^Lu]Lu-DOTATOC product vials or [^177^Lu]Lu-DOTATATE delivery vials containing 15 ml of ^177^Lu in solution and (iii) 30 ml syringes containing a volume of 15 ml for patient administration of [^177^Lu]Lu-DOTATOC or [^177^Lu]Lu-DOTATATE. Ca-DTPA was added to [^177^Lu]LuCl_3_ as a chelator. The solution was transferred from one geometry to the other, and remnant ^177^Lu in one geometry was taken into account before calibration of the next geometry. In 2018–2019 we moved to new hospital facilities and introduced five Capintec CRC-PC Smart Chambers, which were calibrated to the same reading as the Veenstra dose calibrators for a product vial and a syringe (with a homemade plastic insert in the chamber liner to center the tip of the syringe). For each of the geometries we adopted a common calibration factor for all Veenstra or all Capintec dose calibrators, respectively. Uncertainties due to the precision of the reference source, variation between dose calibrators of the same type and variation over time were evaluated.

The activity of Lutathera was measured using the ITG-based calibration factors. A comparison with a reference available from NPL (National Physics Laboratory, UK) was also performed.

For the data analysis in this paper, the activity measured with reference to the planned injection time was decay corrected to the actual injection time (*t*_½_ = 6.647d [34]).

#### Quantitative SPECT (QSPECT)

The SPECT/CT scanners used for the dosimetry scans were two Siemens Symbia T16 SPECT/CT scanners (Siemens Medical Solutions Inc., USA) and from September 2018 also a Siemens Symbia Intevo Bold SPECT/CT scanner, all equipped with two detectors with 3/8" NaI(Tl) scintillation crystals.

##### Calibration and deadtime

Calibration was performed following the procedure described in detail by Beauregard et al. [35] for a similar Siemens SPECT/CT scanner. SPECT scans were performed using medium-energy low penetration collimators, photo-peak at 208 keV (187.2–228.8 keV), lower scatter window 156.0–187.2 keV and general scatter windows covering the range 22.0–156.0 keV, 64 views (32 per detector) in step-and-shoot mode with auto-contouring and 128 × 128 matrix (zoom = 1) with 4.8 mm pixel size. CT was performed using 110 kV voltage, quality reference 60 mAs, collimation 16 × 1.2 mm, pitch 1.0, rotation time 0.6 s and for attenuation correction reconstruction with B08 filter, 5.0 mm slice thickness and 500 mm or 650 mm radial diameter FOV (field-of-view). The SPECT iterative reconstruction protocol was build using Siemens OSEM Flash 3D with 4 iterations, 8 subsets and no post-filtering following Table 3 in Ref. [35] and corrections for attenuation, scatter and collimator blurring.

For the first scanner calibration in 2015, the activity used was in the range 80 MBq to 5.9 GBq. The activity was distributed in eight 1.5 ml microtubes (Eppendorf AG, Hamburg, Germany), which in turn were distributed in three large syringes for easy handling and exchange of total activity. A variable number of syringes were placed between eight bags each containing 500 ml of saline placed in a polystyrene box. The time per view ranged from 20 to 60 s. Twenty-two scans were performed over 30 days on one of the Siemens Symbia T16 scanners (Symbia-1).

The sensitivity *S* and deadtime *τ* was determined from a nonlinear fit (Origin Pro 2015, OriginLab Corporation, USA) of the function *R*_*Wo*_ = *S*·*X*·Exp(-*S*·*X*·*τ*) to data of *R*_*Wo*_ vs*. X,* where *R*_*Wo*_ is the count rate in all the defined energy windows and the scaled activity *X* = *A*·*R*_*Wo*_/*R*_*So*_, where *R*_*So*_ is the total scatter- and attenuation-corrected counts in the SPECT dataset divided by the total acquisition time and *A* is the activity (see Ref. [35] for further details).

The other Siemens Symbia T16 scanner (Symbia-2) had an identical configuration and practically identical performance in acceptance testing following selected parts of the NEMA NU1-2007 protocol [36], and therefore it was anticipated that the same parameters *S* and *τ* could be adopted for Symbia-2. The sensitivity was compared by a few planar and SPECT/CT scans of ^177^Lu sources and furthermore a SPECT/CT scan of the National Electrical Manufacturers Association (NEMA) NU-2 2001 image quality phantom [37] with 6 spheres (inner diameter 10, 13, 17, 22, 28 and 37 mm) filled with a ^177^Lu solution of 2.0 MBq/ml and non-radioactive water in the background volume and additionally a 100 ml plastic bottle filled with 99 ml of the ^177^Lu solution. These acquisitions furthermore served to verify absolute quantification of activity. Analysis of the total activity in the spheres was performed by placing spherical VOIs with diameter equal to the sphere diameter plus 15 mm over the center of the spheres in order to include essentially all counts from the respective spheres.

Following the installation of the Intevo scanner and the move of the two Symbia T16 scanners in 2018, calibrations, as described above, were made of all scanners using 80–6380 MBq (Intevo)/70–3190 MBq (Symbias) of ^177^Lu.

##### Recovery

The NEMA phantom acquisitions were furthermore used to determine the recovery coefficients, RC, for the various sphere sizes, *i.e.* the ratios between activity measured within the spheres to the total activity within the large spheres mentioned above [26]. A fit to these data of the two-parameter function [38]

1 $$\mathrm{RC}\left(d\right)=\frac{1}{1+{\left(\frac{\alpha }{d}\right)}^{\beta }}$$

where *d* is sphere diameter, enabled an extrapolation to volumes typical of kidneys.

##### Stability

In order to document the stability of the scanners, a static planar acquisition of a standard (100–700 MBq, 15 ml in a 30 ml syringe) was performed in fixed geometry on the relevant scanner(s) every day a patient scan was performed.

##### Patient imaging

For the patient scans, the time per view was 40 s at Day 1 and 60 s at Day 4 and 7. The reconstructed SPECT dataset was multiplied by a conversion coefficient *K*, which converted the dataset into activity concentration in units of 100 Bq/cm^3^; a unit chosen in order to exploit the 16 bit range of the images. In case of pixel saturation a scaling of the raw projection images by a factor of 0.1 was applied, and the units of the reconstructed dataset therefore became kBq/cm^3^, when the same conversion coefficient *K* was applied [35].

Deadtime correction was initially performed for every SPECT/CT acquisition, but the correction to the absorbed dose was found to be very small. In order to simplify the reconstruction process, the deadtime correction was later omitted and a general absorbed dose correction was introduced being estimated from a subset of 28 patient data sets. The largest and smallest correction of the absorbed dose was determined, and from this a deadtime correction factor (C_DT_) and associated standard uncertainty was estimated to cover the observed range of corrections to the absorbed dose.

#### Kidney delineation and partial volume correction

**Fig. 2:**
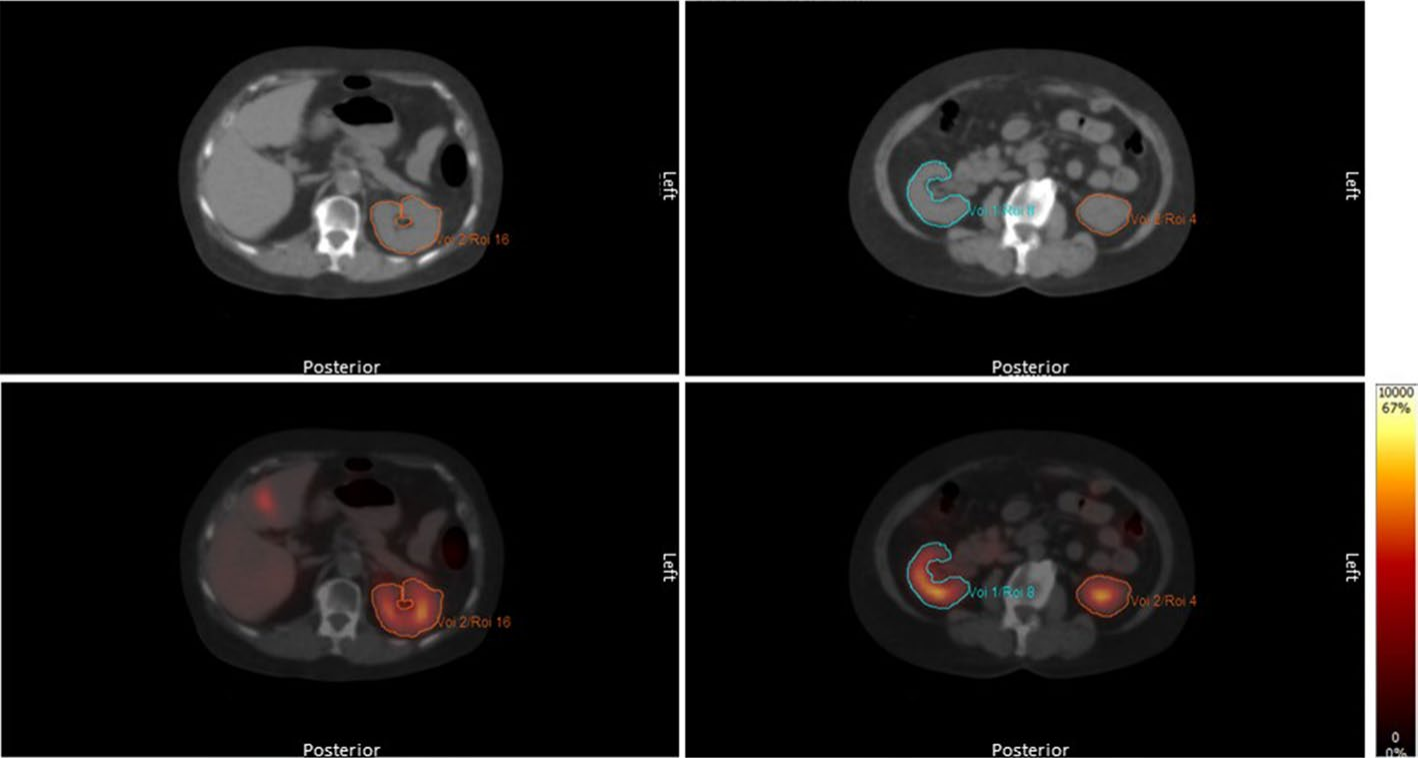
Delineation of kidneys on two transverse slices of a CT scan (top row) and copy to fused images (bottom row) with activity concentration given by the colored scale in units of 100 Bq/cm^3^

The kidneys were delineated manually in Hermes Hybrid Viewer (Hermes Medical Solutions AB, Sweden) by drawing on each transverse slice of the CT reconstruction for attenuation correction. The delineation followed the boundary of the kidneys (Fig. 2) and was copied to the quantitative SPECT reconstruction. The mean of the voxel values, *i.e.* the mean activity concentration, in the delineated volume was then used to generate a time-activity curve for each kidney.

Delineation was performed by a small team of trained technologists or a medical physicist. The delineation was checked by the medical physicist. Before inclusion in the team, each technologist was instructed by the medical physicist and practiced on a small dataset of 2–5 patients. The results (mean concentration and absorbed dose to kidneys) were compared to the results of the other team members.

Some of the activity in the kidneys appeared outside the delineated volume due to partial-volume effects [21], but also due to respiratory [39, 40] or other motion resulting in non-perfect overlap between SPECT and CT. These effects are in reality both patient- and examination dependent but were corrected for by applying a fixed correction factor C_PVC_. C_PVC_ was estimated with an idealized motionless case as a starting point (C_PVC_ ~ 1/RC), *i.e.* using that the recovery coefficient for the kidneys is generally similar to that of a sphere of the same volume [41] and by extrapolating RC to observed kidney volumes using Eq. (1). Finally, a motion-conditioned decrease of RC (increase of C_PVC_) was introduced. This was assumed to be equal to the ratio between respiratory motion and the kidney long-axis, which corresponds to the volume-fraction being shifted for a kidney with constant cross-section along the long-axis. The standard uncertainty was chosen such that C_PVC_ > 1 was fulfilled within the range of ± 6^0.5^ times the standard uncertainty.

#### Time-activity curve and absorbed dose

For 3-SPECT fractions (treatment fractions with SPECT/CT at Day 1, 4 and 7) a time-activity curve was generated as the mean activity concentration *C* for each kidney versus time between end of injection and start of SPECT scan. The data were fitted to a single-exponential decay

2 $${C\left(t\right)=C}_{0}\cdot {e}^{-\lambda t } ,$$

where *C*_*0*_ is the initial concentration and λ the effective decay rate. The number of decays per volume of kidney tissue is equal to the area under the time-activity curve (AUC: area-under-curve). The nonlinear fit and calculation of AUC = *C*_*0*_/*λ* and its uncertainty was performed using Origin Pro 2015 (OriginLab Corporation, USA), and the determined values transferred to a spreadsheet (Microsoft Excel 2010 or 2016, Microsoft Corporation, USA) for dose calculation. Extrapolation of the exponential decay to before the first and after the last datapoint introduced an uncertainty to the total absorbed dose [42]. This was assessed by considering the fractional contribution of these periods, which put a limit on the uncertainty of the total absorbed dose. Further, the study by Delker et al*.* [43] was considered, where they studied in detail the early phase and found minor contributions of 0.6% each from a linear uptake phase, coinciding with the infusion period, and a rapid decay phase. In the late phase, a continuation of the single-exponential decay was expected.

For 1-SPECT fractions (treatment fractions with SPECT/CT at Day 1 only) with a single data point *C*(${t}^{\prime}$), the AUC was determined as

3 $${\text{AUC}}=\frac{C\left({t}^{{\prime}}\right)}{\lambda }\cdot {e}^{\lambda {t}^{\prime}} ,$$

where λ is taken as the effective decay rate of a 3-SPECT fraction in the same treatment series. The relative uncertainty derived from the uncertainty of λ is

4 $$\frac{\text{s(AUC)}}{\text{AUC}}=\frac{{\text{s}}(\lambda )}{\lambda }\cdot {\text{Abs}}\left[\left(1-\lambda \cdot {t}^{{\prime}}\right)\right] .$$

The variation of the effective half-life throughout a treatment series was evaluated by calculating the standard deviation of the ratio between the effective half-life at the first and the fourth treatment fraction using the data given by Garske et al. [44], and this standard deviation was taken as the standard uncertainty due to the assumption of equal effective half-life.

The absorbed dose to each kidney from a 1-SPECT or 3-SPECT fraction was finally calculated by multiplication of the AUC with the mean absorbed energy per decay and division by the tissue density. The absorbed energy has contributions from electrons, gamma self-absorption and cross-irradiation from ^177^Lu in other organs or tumors. For the electron contribution we assumed total absorption of beta radiation, Auger electrons and internal conversion electrons within each kidney (max. electron range 1.6 mm in water) [45]. A value of 0.1479 MeV for the mean energy per decay was adopted from ICRP Publication 107 [46]. The self-absorption of gamma radiation from ^177^Lu activity in each kidney was taken into account by multiplication with a factor of 1.05 based on the Monte Carlo simulation by Hippeläinen et al. [47]. The density of kidney tissue was taken as 1.04 g/ml [48]. Cross-irradiation from other organs or tumors were not accounted for as this was very patient-specific.

Uncertainties of these factors were estimated from computed values for gamma- and beta-radiation absorption for ellipsoidal volumes [49, 50]. We assumed ellipsoids with semi-axes 4.5 cm, 1.5 cm and 5.5 cm [48], computed the effective radius *ρ* defined by Amato et al. [49, 50] (*ρ* = 3V/S, where *V* is volume and S is surface area), scaled it to the minimum and maximum observed kidney volume and computed the absorbed fractions of radiation within these limits. Reported values of mean energy [34, 46, 51, 52] were considered to estimate its uncertainty or possible error. The most recent value was found by summing the average energy emitted from beta-, conversion electron- and Auger-emissions as given by the National Nuclear Data Center at Brookhaven National Laboratory [51] following the recent update of the comprehensive data evaluation by Kondev [34, 52]. The kidney composition [48, 53], and therefore density, is patient specific and a small study of its variation was considered [54] together with another reported value for kidney density [55].

In total, the AUC was multiplied by a factor of 1.95 mGy·ml/(kBq·d) to account for the absorption of beta radiation, Auger electrons and internal conversion electrons in kidney tissue and a factor of 1.05 to account for gamma radiation self-absorption. Further, as mentioned above a partial volume correction factor C_PVC_ and a deadtime correction factor C_DT_ was applied (*C*_DT_ = 1 if deadtime correction was already included in the QSPECT reconstruction).

In total the absorbed dose *D* was calculated as

5 $$D = 1.05 \times 1.95\frac{{{\text{mGy}} \cdot {\text{ml}}}}{{{\text{kBq}} \cdot {\text{d}}}} \times {\text{AUC }} \times C_{{{\text{PVC}}}} \times C_{{{\text{DT}}}}$$

### Uncertainty budget

The standard uncertainties were collected in an uncertainty budget. Combined uncertainties were obtained by adding the uncertainties in quadrature valid for independent error sources.

### Patient dosimetry

Absorbed doses are reported from 3-SPECT fractions only, in order to exclude the uncertainty of 1-SPECT fractions related to the assumption of equal effective half-life. For patients who switched nephroprotection or treatment drug the equal effective half-life assumption was questionable, and in these cases a 1-SPECT fraction was replaced by a 3-SPECT fraction, meaning that some patients were represented by two 3-SPECT fractions in a treatment series of four fractions.

Of 113 treated patients, 3 patients were excluded due to lack of compliance for 3-SPECT fractions, 1 patient due to metal artefacts on CT slices containing part of the kidneys, 1 patient due to cysts near both kidneys resulting in large delineation uncertainty and 1 patient with one kidney only. 139 3-SPECT fractions were performed on the remaining 107 patients; of these fractions one were excluded due to extravasation [56] and 23 due to body parts (mostly arms) outside the CT field-of-view in transverse slices containing part of the kidneys, as this would lead to insufficient attenuation correction. Of the remaining 115 3-SPECT fractions, 59 were treatments with [^177^Lu]Lu-DOTATOC and 56 with [^177^Lu]Lu-DOTATATE (Lutathera®). 20 patients were represented with two 3-SPECT fractions, and of these 12 patients were treated with both [^177^Lu]Lu-DOTATOC and [^177^Lu]Lu-DOTATATE. 2 patients were represented with three 3-SPECT fractions of [^177^Lu]Lu-DOTATOC and [^177^Lu]Lu-DOTATATE, respectively.

Absorbed doses to both kidneys with uncertainties are reported. The specific absorbed doses averaged over the two kidneys are presented for [^177^Lu]Lu-DOTATOC and [^177^Lu]Lu-DOTATATE treatments separately.

Intra-patient comparisons were made for the 22 patients represented with two or three 3-SPECT fractions in order to study the difference between the two treatment drugs. The ratios between specific absorbed dose, effective half-life and specific uptake (*C*_0_/injected activity) for a [^177^Lu]Lu-DOTATATE to a [^177^Lu]Lu-DOTATOC treatment are presented. The same ratios for consecutive [^177^Lu]Lu-DOTATATE or [^177^Lu]Lu-DOTATOC treatments are also presented to illustrate the variation for treatments with the same drug.

## Results

### Dosimetry procedure and uncertainty estimates

#### Dose calibrator calibration and activity measurement

The ^177^Lu reference source was delivered with a certificate stating an overall uncertainty of ±5% on the activity, and assuming it was given with a coverage factor [33] of 2, the standard uncertainty was 3% (rounded up). For each of the geometries we found agreement between the dose calibrators within a 3% range and a 1% range for the Veenstra and the Capintec dose calibrators, respectively. All dose calibrators were stable within ±1% over a year according to measurements of standard sources of Co-57, Co-60 and Cs-137. A standard uncertainty of 1% was included to account for the variation between the individual dose calibrators following the calibration procedure and an additional 1% to account for variation over time, using the triangular distribution assumption.

When receiving deliveries of [^177^Lu]Lu-DOTATATE (Lutathera®; activity stated with overall uncertainty of ±10%) we consistently measured about 10% (4–14%) above the stated activity. In comparison with the NPL reference source (expanded uncertainty 1% with coverage factor 2) we measured 4% higher than the stated activity. Both comparisons showed agreement within the combined uncertainties of the sources.

#### Quantitative SPECT (QSPECT)

##### Calibration and deadtime

**Fig. 3:**
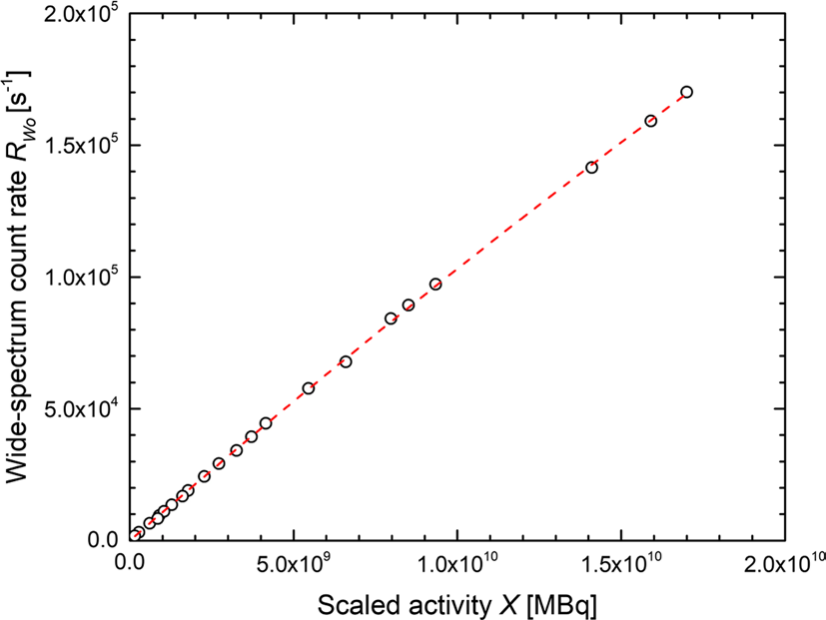
Data and fit for calibration of Siemens Symbia T16 scanner (Symbia-1)

The data for the first calibration of a Siemens Symbia T16 scanner (Symbia-1) are shown in Fig. 3. The fit yielded the sensitivity *S* = (1.081 ± 0.006) × 10^–5^ s^−1^ Bq^−1^ and the dead time constant *τ*  = 0.44 ± 0.04 μs.

The later calibrations of all scanners yielded identical calibration factors within their uncertainties. The values found were *S* = (1.09 ± 0.02) × 10^–5^ s^−1^ Bq^−1^ and *τ* = 0.5 ± 0.3 μs for Symbia-1, *S* = (1.11 ± 0.03) × 10^–5^ s^−1^ Bq^−1^ and *τ* = 0.6 ± 0.4 μs for Symbia-2 and for the Intevo scanner *S* = (1.15 ± 0.05) × 10^–5^ s^−1^ Bq^−1^ and *τ* = 0.7 ± 0.2 μs. Given the agreement between sensitivity and dead time on all scanners, uncertainty-weighted means of the parameters with *S* = (1.104 ± 0.018) × 10^–5^ s^−1^ Bq^−1^ and *τ* = 0.61 ± 0.16 μs were adopted for all scanners after the move. Hence from the calibration we find a 2% standard uncertainty due to *S*, while the relatively large uncertainty on *τ *translates into only 1% standard uncertainty on the absorbed dose through the deadtime correction.

The conversion coefficient *K* was 3.2758 (Day 1) or 2.1839 (Day 4 and Day 7) before the relocation of the scanners and *K* = 3.2090 and *K* = 2.1393, respectively, after the move.

Comparison of Symbia-1 and Symbia-2 using planar and SPECT/CT acquisitions of Lu sources showed agreement within 4%. The SPECT/CT scan of the NEMA phantom showed agreement between Symbia-1 and Symbia-2 within 2% for the total activity in the 5 largest spheres and the plastic bottle.

The total activity was reproduced to 98.9% (Symbia-1) and 98.6% (Symbia-2) for the largest sphere, while for the plastic bottle only 89% of the total activity was found. For two glass vials with 145 MBq and 302 MBq of ^177^Lu scanned on Symbia-2 a deviation of +15% and −3%, respectively, was found in reproducing the known activity with the vials in the calibration phantom, and +4% and −11%, respectively, with the vials outside the phantom. The range of deviations observed (26%) translated to a standard uncertainty of 6% using the triangular distribution assumption.

##### Recovery

**Fig. 4:**
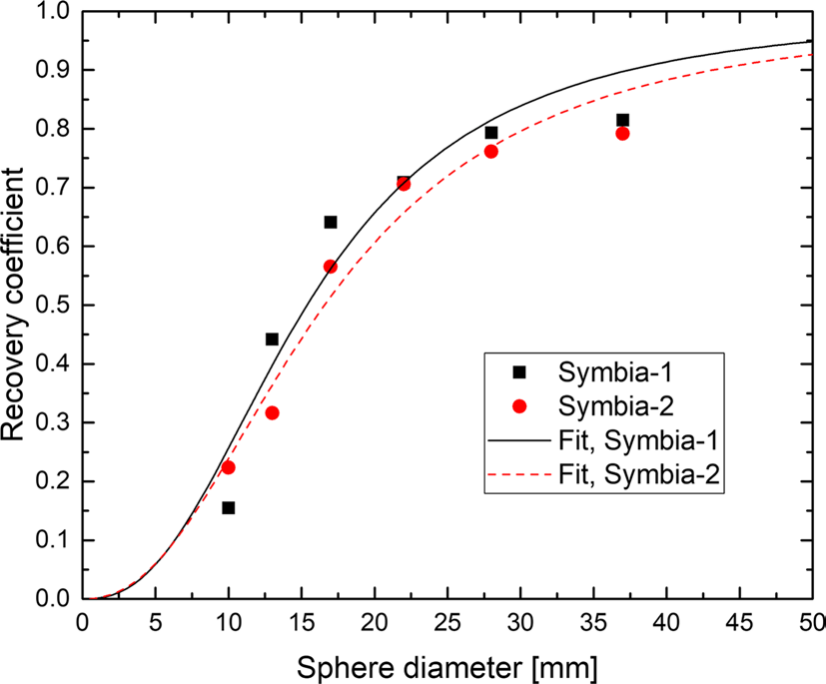
Recovery coefficients for the spheres in the NEMA NU-2 2001 image quality phantom [37] for Symbia-1 and Symbia-2 and fitted curves of Eq. (1)

In Fig. 4 the recovery coefficients are shown for both Symbia scanners with a fit of Eq. (1), which yielded the parameters (*α*; *β*) = (2.47; 15.4) and (2.30; 16.6) for Symbia-1 and Symbia-2, respectively. Extrapolation to the range of observed kidney volumes (60–255 ml) yielded RC of 0.94–0.98 (Symbia-1) and 0.92–0.97 (Symbia-2) with values of 0.97 and 0.96, respectively, at 150 ml.

##### Stability

**Fig. 5:**
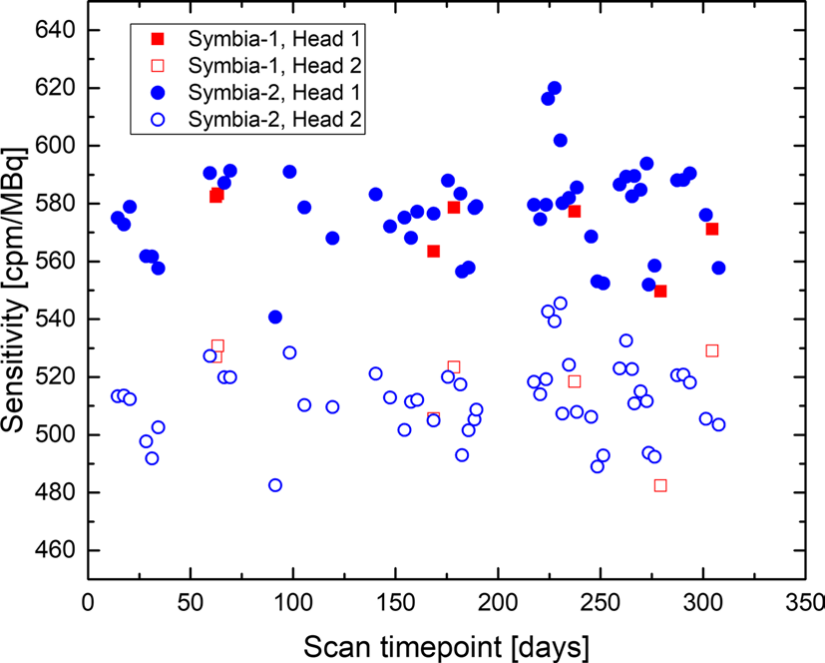
Sensitivity as determined from static images of a ^177^Lu standard. Head 1 was always above the patient board, while head 2 was below and therefore the sensitivity was reduced due to attenuation in the patient board

The sensitivity over a period of 10 months is shown for both Siemens Symbia T16 scanners in Fig. 5. The relative standard deviation of the sensitivity for each detector head was 2.1–3.4%. The variation was in part due to the activity measurement of the varying standards, as apparent ‘jumps’ in sensitivity could be seen when the standard was exchanged (not indicated in Fig. 5) and therefore a standard uncertainty of only 2% was assumed for the stability.

##### Patient imaging

For the subset of 28 patients, the largest deadtime correction of a single time point (Day 1) was 3.4%, while it was smaller at Day 4 and Day 7, and the resulting correction to the absorbed dose was only 2% for this case. The smallest correction to the absorbed dose was 0.5%. The general deadtime correction factor was chosen as *C*_DT_ = 1.01 with an estimated standard uncertainty of 1%, which covered the observed range of corrections using the triangular distribution assumption.

#### Kidney delineation and partial volume correction

In kidney delineation, the deviation from the mean among team members was up to 5% in the mean activity concentration at a single time point and up to 4% in the absorbed dose. The relative standard deviation of the absorbed dose, averaged over the 5 patients in the training sample material, was 2.3%, and hence we include 3% standard uncertainty due to kidney delineation.

For the observed kidney volumes of 60–255 ml, the recovery coefficient extrapolated from the data in Fig. 4 was 0.92–98, yielding on average *C*_PVC_ ~ 1/0.95 = 1.05. The motion-conditioned decrease of RC was found to be about 10%, using values of 1 cm for respiratory motion (mean values of 0.75 cm [39] and 1.11 cm (cranio-caudal direction) [40] have been reported) and a kidney-long axis of 10 cm [48]. This resulted in *C*_PVC_ = 1/0.85 = 1.18. Using the triangular distribution assumption, the standard uncertainty had an upper limit of 7% (*C*_PVC_ > 1), and it was not likely to be any smaller given the variation of RC with volume, the range of kidney motion (standard deviation 4.8 mm and range 2.0–20.5 mm is stated in Ref. [40]) and the simplicity of the motion-conditioned correction. Hence we took 7% as standard uncertainty on *C*_PVC_.

#### Time-activity curve and absorbed dose

**Fig. 6:**
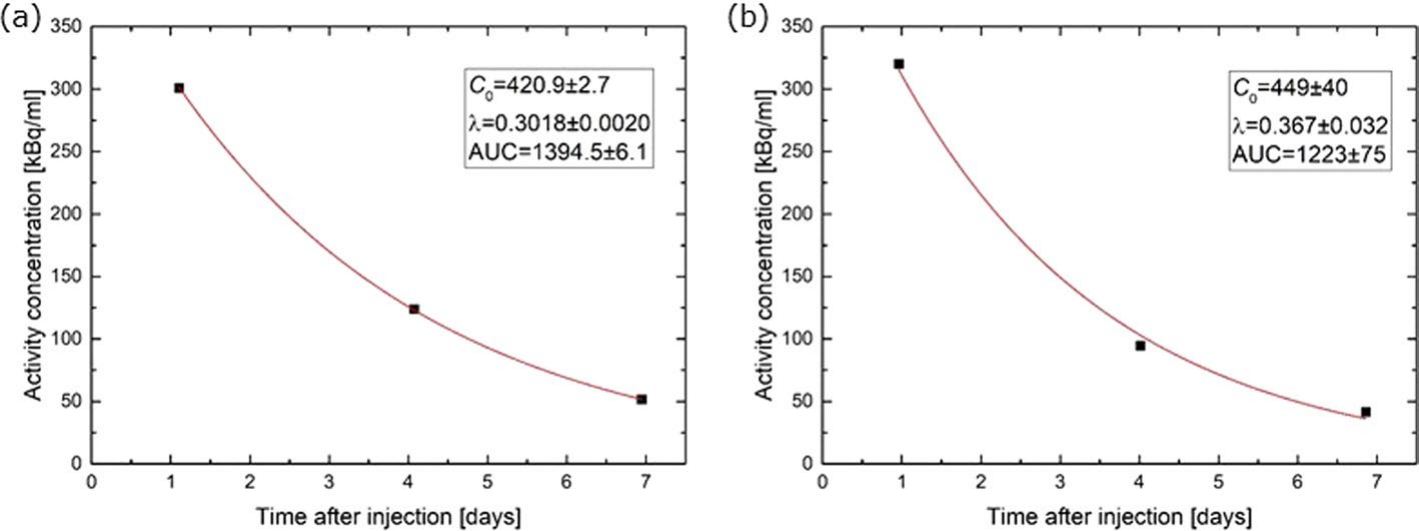
Examples of activity concentration in the right kidney of two patients with **a** excellent and **b** fair agreement with an exponential decay

Two examples of time-activity curves with fits are shown in Fig. 6. In Fig. 6a the effective half-life is 2.3 days, while the relative uncertainties of *C*_*0*_, *λ* and AUC are 0.64%, 0.66% and 0.44%, respectively. In Fig. 6b the effective half-life is 1.9 days and the relative uncertainties of *C*_*0*_, *λ* and AUC are equal to 8.9%, 8.7% and 6.1%, respectively. These are examples which serve to illustrate the variable standard uncertainty of AUC, which typically was 1–10%.

With respect to the uncertainty introduced by extrapolating the exponential decay to before the first and after the last datapoint, the fractional contribution to the AUC before Day 1 is 27% (for the effective half-life mean of 2.2 d, see patient dosimetry data below) and 11% after Day 7. Hence, even the smallest stated standard uncertainty of 1% on the *total* absorbed dose would correspond to about 4% standard uncertainty for the pre-Day 1 phase and about 9% for the post-Day 7 phase. The initial linear phase found by Delker et al*.* [43] was considered negligible here, as the injection was performed in only 5–10 min including flushing with saline as opposed to the 30 min infusion time in Ref. [43]. A contribution of about 0.6% [43] due to the initial rapid decay phase must, however, be expected here. In total 1% standard uncertainty due to extrapolation in the early phase seemed adequate. Given the generally good agreement with a single-exponential decay from Day 1 to Day 7, as exemplified in Fig. 6, a standard deviation of 1% on the total absorbed dose also seemed adequate for the late phase.

For 1-SPECT fractions the standard uncertainty derived from fitting of the 3-SPECT fraction is given by Eq. (4). Taking as an example the data from Fig. 6b and t′ = 24 h, the standard uncertainty was 5.5%. In the data from Garske et al. [44], the ratio between the effective half-life at the fourth and the first treatment fraction [44] has a mean of 0.98 and standard deviation 0.12. This warranted no correction factor to be introduced, but yielded an estimate of the standard uncertainty of 12%.

With respect to absorption of gamma- and beta-radiation, we found the effective radius *ρ* = 2.5 cm for an ellipsoid with semi-axes 4.5 cm, 1.5 cm and 5.5 cm, which had a volume V = 155 cm^3^ [49, 50]. Scaling with *V*^1/3^, the effective radii of a 60 ml and a 255 ml ellipsoid became 1.8 cm and 3.0 cm, respectively. Even for *ρ* = 1.8 cm the absorbed fraction of beta-radiation is >99% [50]. The absorption of gamma-radiation varied by 2.9 percentage points for a 60 to a 255 ml ellipsoid both at 100 keV and 200 keV, representative of the dominant gamma radiation at 113 keV and 208 keV [49]. This variation was translated to a standard uncertainty of 1% using the triangular distribution assumption.

The mean energy per decay is a physical quantity, which, for our purpose, is determined with a negligible standard uncertainty. The most recent value from NNDC is 0.1465 MeV [51].

For the kidney density a value of ‘approximately 1.05 g/cm^3^ ' was reported in ICRP Publication 89 [55] deviating only about 1% from our value of 1.04 g/cm^3^. A standard deviation on the density of about 1% has been reported on a sample of five kidneys [54], which was also assumed here for the standard uncertainty.

### Uncertainty budget

The standard uncertainties found above were collected in the uncertainty budget shown in Table 2, with the total uncertainty given in bold italics. The individual sources contributed with standard uncertainties of 1%-12%, with a few major sources contributing > 5%, namely the reproduction of activity, the partial volume correction, the assumption of equal effective half-life (1-SPECT fractions only), and the uncertainty from the single-exponential fit, which could be significant with typical values of 1–10%. The total uncertainty was 13% for a 3-SPECT fraction and 19% for a 1-SPECT fraction with uncertainties added in quadrature and using fitting values from Fig. 6b as an example.

**Table 2:** Uncertainty budget for the dosimetry procedure

Section in paper	Source	Uncertainty estimate
*Dose calibrator calibration and activity measurement*	Calibration source^a^	3%
	Calibration of dose calibrators^a^	1%
	Dose calibrator stability	1%
*Quantitative SPECT (QSPECT)*	Scanner calibration	2% + 1%
	Reproduction of activity	6%
	Scanner stability	2%
	Deadtime correction^b^	1%
*Kidney delineation and partial volume correction*	Kidney delineation	3%
	Partial volume correction	7%
*Time-activity curve and absorbed dose*	Single exponential model	1% + 1%
	Fitting uncertainty	Individual value from fit (normally 1–10%)
	Assumption of equal effective half-life^c^	12%
	1-SPECT AUC calculation^c^	Individual value dependent on 3-SPECT fit
	Dose factor	1% + 1%
	***Total (3-SPECT—example)***^***d***^	***13%***^***e***^
	***Total (1-SPECT—example)***^***d***^	***19%***^***e***^

^a^For calculations of specific absorbed dose (Gy/GBq) this error cancels out, as it enters in both dose (Gy) measured through scanner calibration and activity (GBq) measured through dose calibrator calibration

^b^If the deadtime correction factor was applied

^c^Only relevant for 1-SPECT fractions

^d^Taking 6% standard uncertainty on AUC as in Fig. 6b. 1-SPECT example: Assuming effective half-life of 2.2 d, scan performed 24 h after injection and 9% standard uncertainty on λ (see Eq. (4))

^e^The stated total uncertainty was obtained by adding the uncertainty components in quadrature and is thus valid for independent error sources. The value was rounded up

### Patient dosimetry

**Fig. 7:**
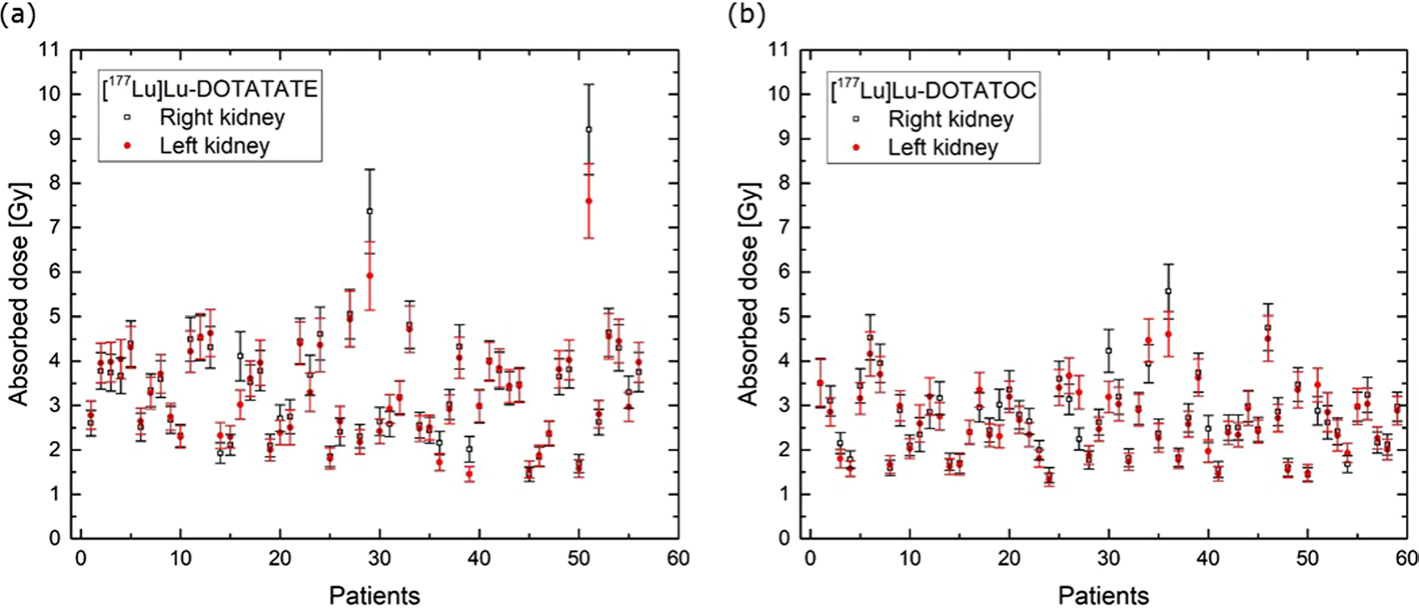
Absorbed doses of both left and right kidneys with uncertainties indicated by error bars for **a** [^177^Lu]Lu-DOTATATE and **b** [^177^Lu]Lu-DOTATOC 3-SPECT fractions

**Fig. 8:**
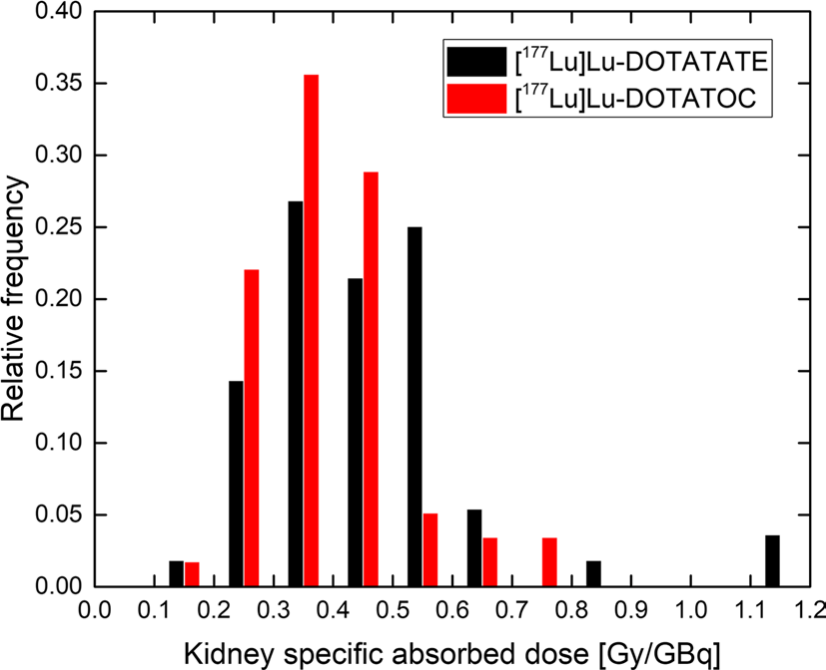
Relative frequency of specific absorbed doses (average over left and right kidneys) for [^177^Lu]Lu-DOTATATE and [^177^Lu]Lu-DOTATOC 3-SPECT fractions

In Fig. 7 the absorbed doses are plotted for (a) 56 [^177^Lu]Lu-DOTATATE and (b) 59 [^177^Lu]Lu-DOTATOC 3-SPECT fractions. The median is 3.3 Gy for [^177^Lu]Lu-DOTATATE (range 1.5–9.2 Gy) and 2.6 Gy for [177Lu]Lu-DOTATOC (range 1.3–5.6 Gy). The observed relative frequency of specific absorbed doses is shown in Fig. 8. For [^177^Lu]Lu-DOTATATE the median is 0.47 Gy/GBq (range 0.20–1.19 Gy/GBq) and the mean 0.46 Gy/GBq, while for [^177^Lu]Lu-DOTATOC the median is 0.37 Gy/GBq (range 0.19–0.78 Gy/GBq) and the mean 0.38 Gy/GBq.

The effective half-life is very similar for [^177^Lu]Lu-DOTATATE (median 2.22 days; range 1.70–3.26 days; mean 2.27 days) and [^177^Lu]Lu-DOTATOC (median 2.13 days; range 1.55–3.21 days; mean 2.19 days).

**Fig. 9:**
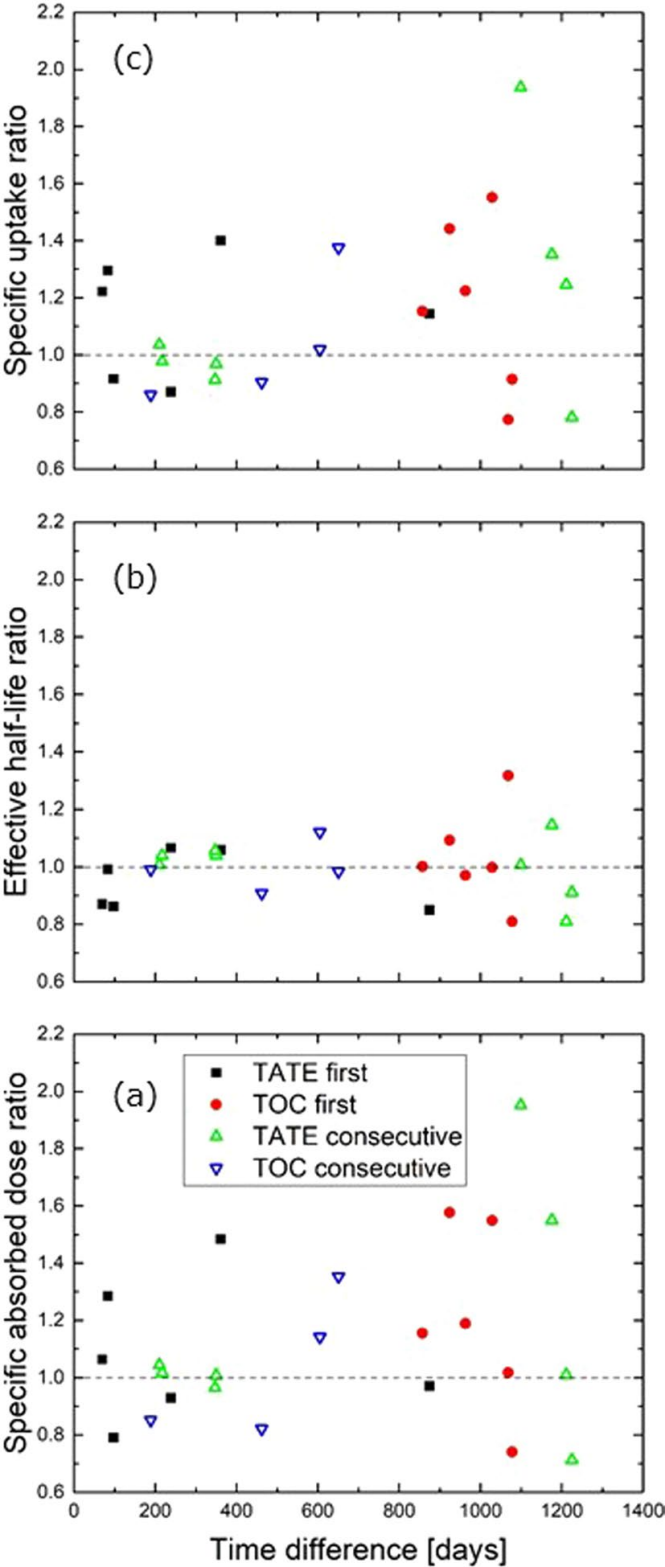
Intra-patient ratios between two treatment fractions vs. time between the fractions for **a** specific absorbed dose, **b** effective half-life and **c** specific uptake. ‘TATE first’: Ratios for a [^177^Lu]Lu-DOTATATE to a [^177^Lu]Lu-DOTATOC treatment, where [^177^Lu]Lu-DOTATATE was given before [^177^Lu]Lu-DOTATOC. ‘TOC first’: As ‘TATE first’, but with [^177^Lu]Lu-DOTATOC given first. ‘TATE consecutive’ and ‘TOC consecutive’: Ratios for consecutive treatments of either [^177^Lu]Lu-DOTATATE or [^177^Lu]Lu-DOTATOC. The dashed lines indicate a ratio of unity

In Fig. 9a we show the intra-patient ratio between the specific absorbed doses with either [^177^Lu]Lu-DOTATATE or [^177^Lu]Lu-DOTATOC given first (‘TATE first’ and ‘TOC first’). In 8 of 12 cases the ratio (‘TATE’/‘TOC’) was above 1, but the difference was not statistically significant (*p* = 0.1, Wilcoxon paired signed rank test). For the effective half-life 5 out of 12 ratios were above 1, while it was 8 out of 12 for the specific uptake. The variation over up to 3 years is also shown for consecutive treatments with the same treatment drug.

## Discussion

### Dosimetry procedure and standard uncertainties

The calibration for quantitative SPECT yielded values which appear reasonable in comparison to the values found by Beauregard et al. [35] using a Siemens TruePoint T6 scanner equipped with a 5/8" NaI(Tl) crystal (*S* = 1.08 × 10^–5^ s^−1^ Bq^−1^ and *τ* = 0.78 μs). The deviations observed in reproduction of activity are about the same magnitude as in the original paper by Beauregard et al. [35]. They performed several tests including a QSPECT patient validation with excellent agreement. Therefore, it is reasonable to assume, also in this work, that the reproduction of activity achieved in patient studies is similar to that obtained in phantom studies. Reproduction of activity in a 100 ml bottle showed a deficit. The bottle was, unlike the spheres, not placed in a scatter medium, and therefore the deficit can most likely be attributed to the attenuation and scatter correction. This difference seems to be in line with the recent work by Frezza et al. [57], where they find a lower sensitivity factor than in their original work [35], and demonstrate that suboptimal scatter correction appears to be the cause of this difference. The deficit observed without scatter medium may indicate the possibility of underestimation of activity in small patients and overestimation in large patients.

Delineation was generally in good agreement within a small group of trained technologists. In our experience, when comparing two delineated volumes, the relative volume difference was larger than the difference in mean activity concentration, as the activity concentration was normally non-zero near the kidney surface, where the two volumes would differ. This makes the delineation procedure less critical to small variations in volume. Delineation was particularly difficult in slim patients, where kidneys were poorly separated from nearby organs. Spill-in from nearby activity in the spleen or in liver metastases was sometimes seen, and could lead to an overestimate of the kidney dose.

The determination of an appropriate factor for correction of partial volume effects is an important but difficult point. We used a fixed correction factor, C_PVC_, but in reality the appropriate factor varies with not only kidney volume and shape, but also motion, which may even vary between examinations of the same patient. The value of up to 0.98, obtained from extrapolation of spheres with volume up to 26.5 ml, can probably not be attained in clinical practice due to motion causing a non-perfect overlap between the kidneys on SPECT and CT. We introduced a 10% correction based on reported values of respiratory motion, however, this was a simplistic ‘order-of-magnitude’ estimate. A model should ideally take into account *e.g.* kidney shape, activity distribution within the kidney, direction of motion and SPECT resolution. With the estimated standard uncertainty on C_PVC_, C_PVC_ is expected to lie in a certain range of 1.01–1.35 (RC = 0.74–0.99), but larger values of C_PVC_ cannot be excluded in case of excessive motion artefacts or if cortex and medulla form a narrow structure, as seen in some patients with small kidneys. In the latter case, the recovery coefficient can be significantly reduced as compared to a sphere or ellipsoid, as demonstrated by the Würzburg group using a 2-compartment dedicated kidney phantom with activity in the cortex only and no background [58]. A value of RC = 0.64 was *e.g.* found for a cortex volume of 100 ml, as opposed to 0.83 for an ellipsoid of the same volume. Further studies are clearly warranted to study the variation of partial volume effects in clinical practice.

The AUC has a relative uncertainty, which is smaller than on both the amplitude *C*_*0*_ and the decay rate λ. This is due to the fact that an increase of *C*_*0*_ is counteracted by an increase of λ; mathematically it follows from Eq. (60) in Ref. [26] and that the covariance *u*(*C*_*0*_, λ) was generally found to be positive.

The assumption of equal effective half-life throughout a treatment series is generally good with standard deviations in the effective decay rate of about 12% between treatment fractions [44], and it has furthermore been shown that the absorbed dose derived from a 1-SPECT fraction compares well with that derived from a 3-SPECT fraction [59]. As noted in Ref. [44], in patients with large changes in tumor load, marginal kidney function or cardio-vascular risk factors larger changes in kidney uptake or clearance can frequently be observed, and therefore repeated 3-SPECT fractions instead of 1-SPECT fractions are recommended. In the present study a 3-SPECT fraction replaced a 1-SPECT fraction, if the treatment drug or nephroprotection was changed within a treatment series.

The factor of 1.95 mGy·ml/(kBq·d) in Eq. (5) has only a small uncertainty or error. The mean energy of 0.1479 MeV enters and is only 1% above the more recent value from NNDC [51], and it is furthermore in good agreement with the value from the Radar website (0.1472 MeV) [60], which enters into the frequently used program Olinda/EXM [61]. Olinda/EXM applies a value of 1.05 g/cm^3^ for the kidney density following Ref. [55], while we use 1.04 g/cm^3^ here. While these are small systematic deviations, some authors use 1.0 g/cm^3^ [44, 62, 63], which yields a more significant, but easily correctable, difference. The product of 1.05 and 1.95 mGy·ml/(kBq·d) equals 2.05 mGy·ml/(kBq·d), which is in excellent agreement with the isotope-specific S-values for the adult kidney model reported in MIRD Pamphlet 19 [48].

For the neglected cross-fire radiation to the kidneys from ^177^Lu in other organs or tumors, an upper limit of about 10% was suggested by Hippeläinen et al. [47] based on a specific example and it was also considered to be below 10% by Sandström et al. [63].

### Uncertainty budget

The combined uncertainties stated for a 3-SPECT and a 1-SPECT fraction are obtained by adding the components in quadrature, which is only correct for independent error sources. An upper limit of the uncertainty in the dosimetry procedure can be found by assuming complete positive correlation between the error sources, which leads to a 33% uncertainty for a 3-SPECT fraction by summing the terms linearly. A detailed discussion of correlations is a topic of its own [26] and beyond the scope of the present paper. The stated total uncertainty, or its bounds, is nevertheless a good estimate of the total uncertainty of the dosimetry procedure, which is useful for a consideration of the relevance and application of the dosimetry procedure. The estimated uncertainty compares reasonably well with the numbers stated in Refs. [24, 26] using similar dosimetry procedures.

The main uncertainty contributions are due to uncertainty in activity quantification and partial volume correction and possibly from the fitting procedure. For a full PRRT treatment series, an additional significant contribution applies due to an assumed effective half-life in 1-SPECT fractions.

### Patient dosimetry

Specific absorbed doses have been reported in the literature by several groups. A fairly recent compilation of results is given in Ref. [19] for [^177^Lu]Lu-DOTATATE, where mean or median values vary from 0.31 to 1.0 Gy/GBq. The lowest value of 0.31 Gy/GBq is exceptional in this compilation as all other values are 0.6 Gy/GBq or above, but similarly low specific absorbed doses of 0.43 Gy/GBq, 0.40 Gy/GBq, 0.29 Gy/GBq and 0.23 Gy/GBq have been reported [64–67]. Our median value of 0.47 Gy/GBq and mean 0.46 Gy/GBq fall well within the range of these reported values. For [^177^Lu]Lu-DOTATOC only a few literature values are to our knowledge available; Schuchardt et al. [16] report median (range) of 0.6 Gy/GBq (0.3–1.6) while Guerriero et al. [68] report 0.7 ± 0.2 Gy/GBq (mean ± standard deviation). Our median value of 0.37 Gy/GBq (range 0.19–0.78) and mean 0.38 Gy/GBq is somewhat lower but not in disagreement with these values. The same authors also compare the specific absorbed dose of [^177^Lu]Lu-DOTATATE and [^177^Lu]Lu-DOTATOC and find a ratio (‘TATE’/‘TOC’) of 1.33 (ratio of medians) and 1.43 (ratio of means), respectively, while we find a ratio of 1.27 and 1.21, respectively.

These intra-patient ratios may, however, be heavily influenced by changes in patient status between the treatments being given up to 3 years apart. Indeed, in the cases where a patient was treated more than once with the same drug, we could observe (Fig. 9a) that the variation in specific absorbed dose tended to increase with time between the treatments, and that the ratios for treatment with two different drugs were of a similar magnitude as for treatments with the same drug.

### PRRT and EBRT

The specific absorbed doses reported in the literature for PRRT vary by up to a factor of 4. Differences in the imaging procedure (planar, SPECT or hybrid [69] and variable use of attenuation and scatter correction) or the dosimetry calibration and calculation procedure may on the one hand introduce a significant variation. It has *e.g.* been shown that dosimetry based on 2D rather than 3D imaging can lead to an overestimate of about 25% [69], or even up to a factor of 2–3 [70]. On the other hand different patient cohorts, differences in the nephroprotective protocols or other differences in the treatment given such as peptide amount used [71–73] or the use of carrier-added or no-carrier-added ^177^Lu [64] will also contribute to this rather large variation on specific absorbed dose. The uncertainties of the reported values are rarely stated, which makes a comparison difficult, and it is generally not possible to say whether this large difference is mainly due to the dosimetry procedure or due to biological differences in the patient cohorts or differences in the treatment procedures.

In comparing dosimetry results from different treatment sites, it is obviously important to note and, when relevant, correct for systematic differences. As noted above the applied kidney density, either directly or through the use of S-values or dose factors [74], may vary up to 5%, while the variation of the mean energy per decay is very small. Different ^177^Lu reference sources for calibration may also introduce a significant difference, about 5% is not unlikely. This transfers to the absorbed dose and *e.g.* reported thresholds for kidney toxicity or other outcomes in similar applications, such as spleen, image-based red-marrow or tumor dosimetry [38, 75–77].

For the dosimetry procedure to be relevant in a clinical or research setting, a reasonable requirement is that the uncertainty of the procedure is not significantly higher than the uncertainty of the EBRT derived absorbed dose limit. Furthermore, the dose limit for kidneys is currently not well established in PRRT, and any dose limit obtained in a PRRT study with acceptable precision would add to our current knowledge [19].

A TD_5/5_ limit (the radiation dose that would result in 5% probability of complication within 5 years from treatment) for whole-kidney irradiation of 23 Gy adopted directly from EBRT [78] has frequently been quoted, however this limit is probably too cautious. The low dose-rate in PRRT (~ mGy/min) results in less DNA double strand breaks and allows for increased repair when compared to EBRT (dose rate ~ Gy/min). This can be taken into account by applying the linear-quadratic (LQ) model to calculate the biologically effective dose (BED) of a PRRT treatment, which in turn can be compared to the BED of a given EBRT fractionation scheme [12, 79–81]. Furthermore, the inhomogeneous dose distribution in PRRT is expected to increase the dose limit in PRRT as compared to EBRT [82].

The limit of 23 Gy is stated in the work by Emami et al. [78] with a 5-week fractionation schedule. Dawson et al. [83] states 18–23 Gy in 0.5–1.25 Gy fractions, while Marks et al. [84] states 15–18 Gy in 1.8–2.0 Gy fractions. The biologically effective doses of EBRT schemes with 23 Gy in 25 fractions or 18 Gy in 9 or 10 fractions are 31.5–33.0 Gy, using *α*/*β* = 2.4 Gy in the LQ-model [85]. Hence these fractionation schemes are in very good agreement, but the absolute value of the BED may vary by about 10% due to about 20% variation in *α*/*β* values [86].

The BED from a PRRT treatment series has two terms, the absorbed dose and a term quadratic in absorbed dose. The latter involves α/β and additional parameters, the repair time of sub-lethal damage and the patient-specific effective half-life [79, 85]. The absorbed dose is the dominant term, but the quadratic term contributes with additional uncertainty to the BED, which depends on the magnitude of the term and the uncertainty of the model parameters. In total, we find that the uncertainty of the BED of a PRRT treatment series is at least a factor of 2 larger than the variation of BED in EBRT schemes, including the uncertainty of *α*/*β*. Given the complexity of dosimetry after PRRT this seems like an acceptable number, and the dosimetry appears to be useful in a clinical or research setting.

## Conclusion

The presented kidney dosimetry procedure yields specific absorbed dose values in agreement with values from other treatment sites, towards the lower end of reported values. The estimated uncertainty of the procedure is comparable to numbers reported using similar dosimetry procedures. The greatest reduction in uncertainty can be obtained by improved activity determination, partial volume correction and by performing more than a single post-treatment SPECT/CT scan after all treatment fractions. With the level of uncertainty estimated, the presented kidney dosimetry procedure appears to be relevant for use in a clinical or research setting.

## Data Availability

The datasets used and/or analyzed during the current study are available from the corresponding author on reasonable request.

## Abbreviations

AUC: Area-under-curve
BED: Biologically effective dose
DTPA: Diethylenetriaminepentaacetic acid
EBRT: External beam radiation therapy
EDTA: Ethylenediaminetetraacetic acid
GFR: Glomerular filtration rate
NEMA: National Electrical Manufacturers Association
NET: Neuroendocrine tumor
OSEM: Ordered subset expectation maximization
PRRT: Peptide receptor radionuclide therapy
QSPECT: Quantitative SPECT
RC: Recovery coefficient
